# Weld Magnification Factor Approach in Cruciform Joints Considering Post Welding Cooling Medium and Weld Size

**DOI:** 10.3390/ma11010081

**Published:** 2018-01-05

**Authors:** Oscar Araque, Nelson Arzola

**Affiliations:** 1 Departamento de Ingeniería Mecánica, Universidad de Ibagué, Ibagué 730001, Colombia; 2 Department of Mechanical and Mechatronics Engineering, National University of Colombia, Bogota 111321, Colombia; narzola@unal.edu.co

**Keywords:** cruciform joint, fatigue, semi-elliptical crack, cooling, weld magnification factor, fracture mechanics

## Abstract

The objective of this research is to develop an experimental-theoretical analysis about the influence of the cooling medium and the geometry of the welding bead profile in fatigue life and the associated parameters with structural integrity of welded joints. A welded joint with cruciform geometry is considered using SMAW (Shielded Metal ArcWelding), plates in structural steel ASTM A36 HR of 8 mm of thickness, and E6013 electrode input. A three-dimensional computational model of the cruciform joint was created using the finite element method. For this model, the surface undulation of the cord and differentiation in the mechanical properties of the fusion zone were considered, the heat-affected zone (HAZ) and base material, respectively. In addition, an initial residual stress field, which was established experimentally, was considered. The results were a set of analytical expressions for the weld magnification factor M_k_. It was found that values for the latter decrease markedly in function of the intensity of the cooling medium used in the post welding cooling phase, mainly due to the effect of the residual compressive stresses. The obtained models of behavior of the weld magnification factor are compared with the results from other researchers with some small differences, mainly due to the inclusion of the cooling effect of the post weld and the variation of the leg of the weld bead. The obtained analytical equations in the present research for M_k_ can be used in management models of life and structural integrity for this type of welded joint.

## 1. Introduction

One of the common failure phenomena in structural engineering materials is fatigue failure. This is associated with certain flaws in the material or any geometric detail, which, after a certain number of load cycles, generate the initial fatigue crack. Either through manufacturing or created by situations of use, pre-existing flaws create the critical conditions from which the material breakage is developed. Fracture mechanics’ purpose is to analyze and determine the mechanical behavior of structural elements, when considering the existence of flaws in the material to define the conditions or criteria of breakage [1].

The first theory that explains the fracture of cracked solids, known as linear elastic fracture mechanics (LEFM), was initially proposed by Griffith at the beginning of the last century (Griffith, 1920), and subsequently developed by Irwin, in the second half of the same (Irwin, 1957). Up to its appearance, only the failure by plastic collapse, where the material deforms plastically without any fracture, had well-structured physical and mathematical foundations. The LEFM came to fill the gap that existed in the opposite situation of the plastic collapse, when the fracture occurs in conditions of small deformation and in stress levels that are much lower than those that lead to the start of the material plastic deformation processes [2].

Using the principles of the LEFM, it is possible to assess the stable propagation of fatigue cracks in welded joints when using the empirical relationship proposed by Paris and Erdogan [3,4].

(1) $$\frac{\mathrm{da}}{\mathrm{dN}}\;=\;C{\left(\Delta K\right)}^{m}\;=\;C{\left(\beta \Delta \sigma \sqrt{\pi a}\right)}^{m}$$

To:

(2) $$\Delta K_{\mathrm{th}}\leq \;\Delta K\;\leq \;\Delta K_{\mathrm{Ic}}$$

where the C and m parameters are constant of the material for a range of stress Δσ and R (load rate) fixed; ΔK is the range of the stress intensity factor; β is a dimensionless function that depends on the geometry of the component and the crack size (a); $\Delta K_{\mathrm{th}}$ is the range of the stress intensity factor threshold; and, finally, $\Delta K_{\mathrm{Ic}}$ is the fracture toughness of the material for the condition of plane-strain.

When the stress intensity factor reaches a critical value, and the ASTM E399 and ASTM D5045 requirements are met, the critical value can be regarded as a material property called fracture toughness for plane-strain, K_IC_. For that value, crack starts its unstable spread, fracturing the component into two parts. In this way, the local fracture approach on Mode I is determined, on the basis of the following expression [5,6,7].

(3) $${\beta \sigma }_{f}\sqrt{\pi a}\to K_{\mathrm{Ic}}$$

In welded joints, the stress fields in front of the crack are more complex to determine due to the microstructural changes that occur as a result of the thermal cycle of the cooling system [8]. The crack tip in a weld can be described as a semi-elliptical curve with depth (a) and length (2c). In general, using Mode I, the stress intensity factor is given by [9].

(4) $$K_{I}\;=\;Y\sigma \sqrt{\pi a}$$

where σ is the applied stress, and Y is a correction factor dependent on the load and the geometry of the crack size. The Y parameter is influenced by a number of factors that can be represented as follows:

(5) $$Y\;=\;\frac{M_{k}+M_{S}+M_{t}}{\emptyset^{o}}$$

where M_k_ is a factor which considers the presence of the weld; M_S_ is a correction factor of the free surface area near the crack tip; M_t_ is a correction factor of the free surface in the crack tip; and, finally, $\emptyset^{o}$ complete is the integral of the ellipse. The latter can be expressed as:

(6) $$\emptyset^{o}\;=\;\int_{0}^{\pi /2}{\left\{1-\left(1-\frac{a^{2}}{c^{2}}\right){\mathrm{sen}}^{2}\emptyset \right\}}^{1/2}d\emptyset$$

where ∅ is defined as the angle of the ellipse. Values of M_S_ and M_t_ depend on the joint geometry, and failing to evaluate them can lead to an error that is normally about 0.13%. The latter is due to the fact the stress field is low-intensity when the distance is greater from the weld toe. Therefore, it can be avoided [10]. 

A number of researchers have determined expressions for the calculation of M_k_, such as Lie and Zhao, and Maddox and Andrews, who made a review of the British Standards PD6493 and BS7608, for the steel structures cruciform design subjected to fatigue, establishing a value of M_k_ between 0.83 and 1.00 for cracks located at the weld of toe [11,12]. The Hobbacher researcher, found an expression for M_k_, for the case of a cruciform welded joint and 0.02 mm-sized crack, finding that the effect of the weld of toe produces a variation of 5% for various relationships of assessed aspects. The obtained equation by Hobbacher is described below [13]:

(7) $$M_{k}\;=\;C{\left(\frac{a}{T}\right)}^{k}{\;\mathrm{for}\;M}_{k}\geq 1$$

The magnitudes of C and k are dependent on the aspect and the geometry of the joint. Maddox presented a dimensionless factor $M_{k}$, which allows for the estimation of the influence of stresses that are generated by the geometric profile of the welded joints on the stress intensity factor [12].

(8) $$M_{k}=\frac{K}{\sigma \sqrt{\pi a}}$$

The researcher [9,14] carries out a comparative analysis between the estimated models by Maddox, Andrews, and Hobbacher for the determination of the weld magnification factor M_k_. In this work, it is determined that the crack depth is a parameter that affects between 15% and 65% of the parametric equation for the calculation of M_k_. Equally, researcher Brennan [15] developed a comparative parametric equation for the determination of the weld magnification factor, in a cruciform welded joint. In addition, the results were compared with those previously developed by the researchers [10,16,17], establishing a good level of correspondence between the magnitudes encountered and the previous research. In the case of welded joints in test tubes with cruciform geometry, Zhao and Lie [11] include a set of equations for estimating the effect of misalignment on different types of welded joints with a semi-elliptical surface crack. Takeshi shows [18] shows that failures start at the root of the weld being the stress hub that defense the propagation of the crack and its life.

The study using numerical methods of the transient thermal behavior of the welding process goes back to the 1980s, highlighting the work done by Friedman [19]. Among the numerical methods used to carry out the study of transitional period thermal behavior, the finite element method: one of the most popular methods. This technique has gained special importance, mainly when it includes a mesh refinement around the tip of the crack, besides the effect of the thermal cycle in the stress intensity factors K_I_ assessment and the weld magnification factor M_k_. Although, conceptually, the factors are obtained in a direct way, finite element analysis, with conventional elements near the crack tip, underestimates the stress increase in gradient and displacement. Instead of using ever smaller elements, size 1/$\sqrt{r}$, some researchers [20,21] introduced a direct method, by moving the composed node of 8-noded quadrilateral elements up the quarter points in the crack tip and relocating the nodes of the mid-point to a fourth at the end of the crack. In the case of linear elastic deformation, the elements Plane2 (2-D, 6-noded triangle), Plane82 (2-D, 8-noded quadrilateral), and Solid95 (3-D, 20-noded brick), are used in ANSYS [16] to stabilize the residual stress field by moving the nodes to a fourth of the tip of the crack. Once the field of stress is established, the parameters of fracture are obtained [22]. Certain configurations of elements and nodes produce unique displacements. While this type of behavior is undesirable for the majority of the analyses, it is ideal for elasticity problems in cracks. By forcing elements in the crack tip to have a unique deformation, $1/\sqrt{x}$ can improve the accuracy and reduce the need for a high degree of refinement of mesh in the crack tip. This singular deformation is only applied in the crack tip. 

Some contributions of this research are the three-dimensional computational analysis of a welded joint by using the finite element method, Another contribution is the surface weld bead and the differentiation in the mechanical properties of the fusion zone, the heat-affected zone (HAZ), and the base material zone. In addition, the use of an initial residual stress field for the welded joint and adjacent region to emulate the actual experimental model. The fundamentals of Fracture Mechanics were employed in the numerical modeling of the welded joint with the presence of a surface crack semi-elliptical type discontinuity at the weld toe. The latter is defined as a semi-elliptical surface crack with a small aspect. Because of this study, a set of mathematical models for the weld magnification factor were obtained for cruciform welded joints, which can be used in the prediction of the fatigue life of this type of welded joint.

## 2. Materials and Methods

For the definition of the experimental and analytical procedures, previous studies were used as reference in cruciform test tubes that were subjected to biaxial cycles of stress to analyze fatigue. In these studies, the thicknesses, welding dimensions, and size and penetration depth of the weld were observed. For the experimental development of the present work, a carbon steel ASTM A36 HR Commercial 8 mm thickness and material of the electrode E6013 were used. The Shielded Metal ArcWelding (SMAW) process is a simple, low cost and suitable way of joining most metals and alloys commonly used in industry [23]. The electrical characteristics of the process (SMAW) used in the joint are shown in Table 1, for each weld size (leg).

**Figure 1:**
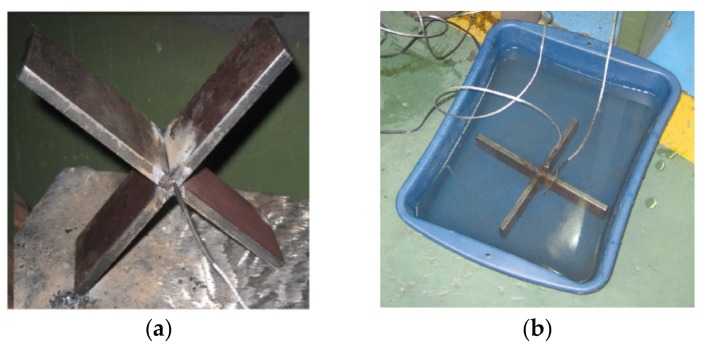
Connection of the thermocouples. (**a**) Welded specimen; (**b**) Connected thermocouples.

For the purpose of generating an experimental input to the simulation runs and compare the residual stress from the thermal cycle [24], the measurement of temperature on the test specimen was carried out. The latter was made during the post welding cooling for the two media (air and water) by using two type K thermocouples. They were located laterally on each edge of the bead welded. The thermocouples were connected to a data acquisition card NI DAQ 9211, mounted on the NI CDAQ-9172, and then to the personal computer. The layout of the thermocouples in the measuring cylinder is shown in Figure 1. LabView Signal Express 2011 software (National Instruments, Austin, TX, USA) was used to acquire and process data from the thermocouples, and to obtain the cooling curves.

**Figure 2:**
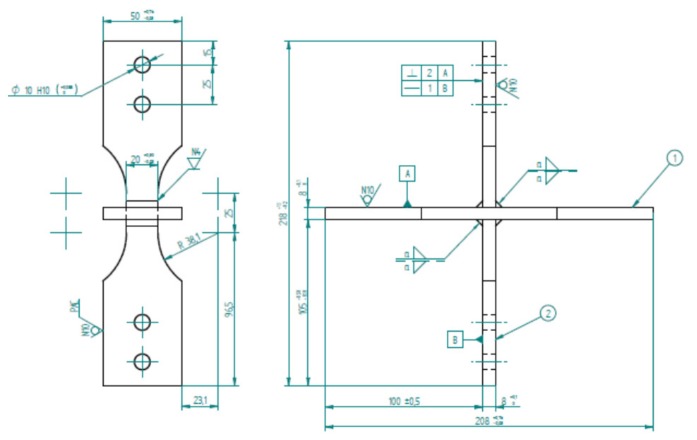
Geometric fixture test (mm scale).

For the process of plate-cutting, the technique of high-density plasma was used. Due to the cutting technique used, the heat affected zone (HAZ), with a thickness of 8 mm, reached a millimeter of depth of the surface that results from the cut. After this preliminary cut, the central area of the test piece went into a mechanical process through milling to remove the endings of the weld bead, prone to higher density of defects product at the beginning, and breakdown of the electric arc. The geometry of the fixture to be used in the tests after final machining is presented in Figure 2.

The variables used for the manufacture of the test specimens are indicated below in Table 2. 

Fatigue tests were performed by axial load on cruciform geometry specimens, for different load ratios (R), defined as:

R = P_min_/P_max_, where; P_min_: Minimum load and P_max_: Maximum load

**Figure 3:**
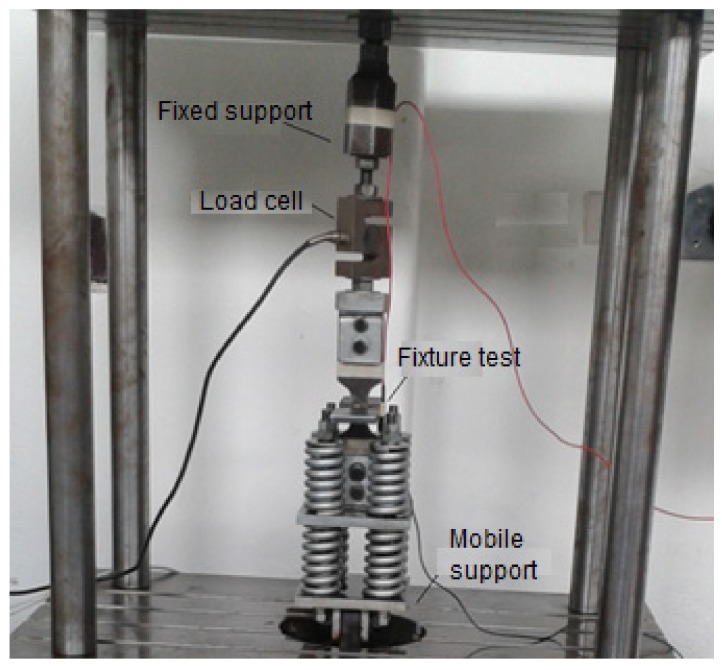
Assembly for the axial fatigue test.

The assembly made for the test is shown in Figure 3.

The operating parameters of the equipment used are indicated in Table 3.

To begin the simulations by the finite element method, the software ANSYS ([16], Swanson Analysis Systems, Inc., Canonsburg, PA, USA) was used. The determination of stress intensity factors for geometries and application modes of simple loads can be carried out through easily implemented analytical solutions. But, when the geometries and loads are more complicated, these induce complex stress and strain fields on the structural component; therefore, it is recommended to use the finite element method to determine said factors [25]. Also, the displacement correlation technique (DCT) is relatively simple to perform and offers sufficient precise solutions for the purpose of this work. Thus, the DCT method is employed in the modeling of the cracks in the weld joint analyzed.

To describe the stress field intensity in the region near the crack vertex, it is necessary to use singular elements, with an additional node at a distance of a quarter of the size for the fissure vertex. With these singular elements, the stress intensity factors can be calculated in the following way.

(9) $$K_{I}\;=\;\frac{\mu }{k+1}\cdot \sqrt{\frac{2\pi }{L}}\cdot \left\{4\left(v_{b}-v_{d}\right)+\left(v_{e}-v_{c}\right)\right\}$$

(10) $$K_{\mathrm{II}}\;=\;\frac{\mu }{k+1}\cdot \sqrt{\frac{2\pi }{L}}\cdot \left\{4\left(u_{b}-u_{d}\right)+\left(u_{e}-u_{c}\right)\right\}$$

With:

(11) $$\mu \;=\;\frac{E}{2\left(1+\nu \right)}\;\;\;k\;=\;\{\begin{array}{c} 3-4\nu \;\left(\mathrm{plane}\;\mathrm{strain}\right)\\ \frac{3-\nu }{1+\nu }\;\left(\mathrm{plane}\;\mathrm{stress}\right) \end{array}.$$

where:

K_I_, K_II_: Stress intensity factors for load modes I and II, respectively (MPa·$\sqrt{m}$).
E: Elasticity modulus of the material (MPa).
ν: Poisson’s ratio of the material.
L: Characteristic length of the singular element (mm).
u_i_; v_i_: Displacements of the nodes of the singular elements (mm).

**Figure 4:**
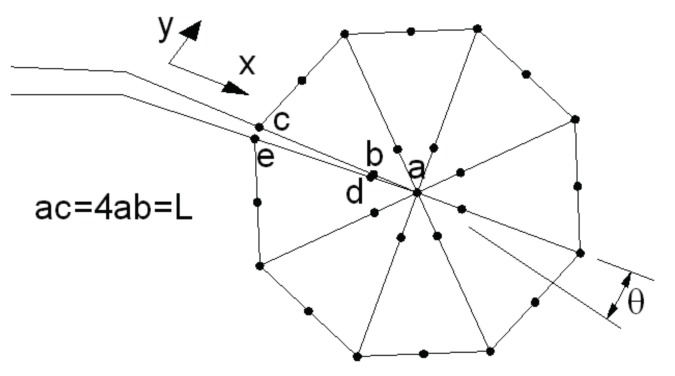
Disposition of control nodes on the crack vertex.

Figure 4 shows the singular elements, the location of the nodes and the displacements employed in calculating the stress intensity factors.

The tip of the crack must be meshed with small singular concentric elements and should not vary in size as the crack extends. The rest of the component is meshed with quadrangular elements that provide adequate precision.

Various methods are available to establish the orientation of the crack as it extends, although they basically lead to similar results. This work uses the strain energy density method on the crack vertex (ψ), which is expressed according to (12). The relative local minimum of ψ corresponds to a large volume change and is identified with the region that is dominated by macro dilatation leading to crack growth. Accordingly, this method establishes that the crack propagates in the direction of minimum strain energy released [26].

(12) $$\psi \;={\;A}_{11}K_{I}^{2}+2A_{12}K_{I}K_{\mathrm{II}}+A_{22}K_{\mathrm{II}}^{2}$$

where: A_ij_: Coefficients that depend on the material’s elastic properties.

**Figure 5:**
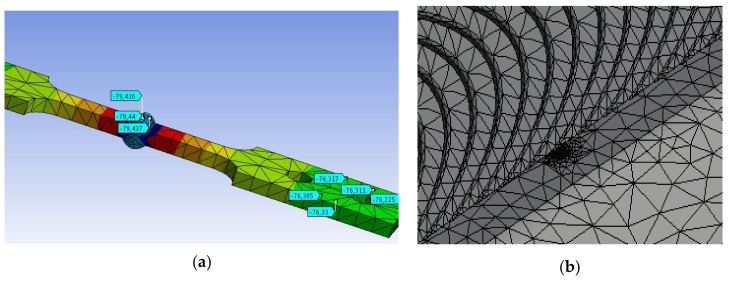
(**a**) The temperature profile for the cooling cycle post welding and residual stress C = 5 mm, (**b**) semi-elliptical crack on welding and plane generated for the finite element model.

A three-dimensional (3D) computer model of the cruciform test tubes for each of the two legs of welding considered included the temperature profiles obtained experimentally and the determination of the profile of stress for the residual cooling conditions in calm air and water, as shown in Figure 5a. At a later stage in the modeling, surface semi-elliptical, a crack was included at the weld toe, as shown in Figure 5b. The interest in this second model focused on studying the stress-strain field near the front of the semi-elliptical crack under various conditions of cyclic loading (changing the load ratio R). Crack sizes used in this work for the computer simulations are shown in Table 4, naming c, the size of the semi-major axis, and a, the dimension of the semi-minor axis of the semielliptical crack.

With the FEM (finite element method) model implemented, the values of the weld magnification factor are determined for crack sizes that appear in Table 2. The weld magnification factor is calculated by:

(13) $$M_{k}\;=\;\frac{{\;K}_{I\;\left(\mathrm{MEF}\right)}}{\sigma_{\mathrm{nom}}\sqrt{\pi a}}$$

where:

$K_{I\;\left(\mathrm{MEF}\right)}$: Stress intensity factor obtained by FEM (MPa·$\sqrt{m}$).
$\sigma_{\mathrm{nom}}$: Nominal stress (MPa).

The magnitudes of the nominal and alternant stress of operation appear in Table 3. It is calculated using the following equation:

(14) $$\sigma_{\mathrm{nom}}=\frac{F}{\mathrm{TL}}$$

Being:

F: Load operation (N).
T: Plate Thickness (mm).
L: Length of the weld bead (mm).

(15) $$\sigma_{\mathrm{alt}}\;=\;\frac{F}{2\mathrm{CL}}$$

Being:

C: weld size leg (mm).

The values of the nominal and alternant stresses of the axial fatigue test are shown in Table 5.

## 3. Discussion and Results

**Figure 6:**
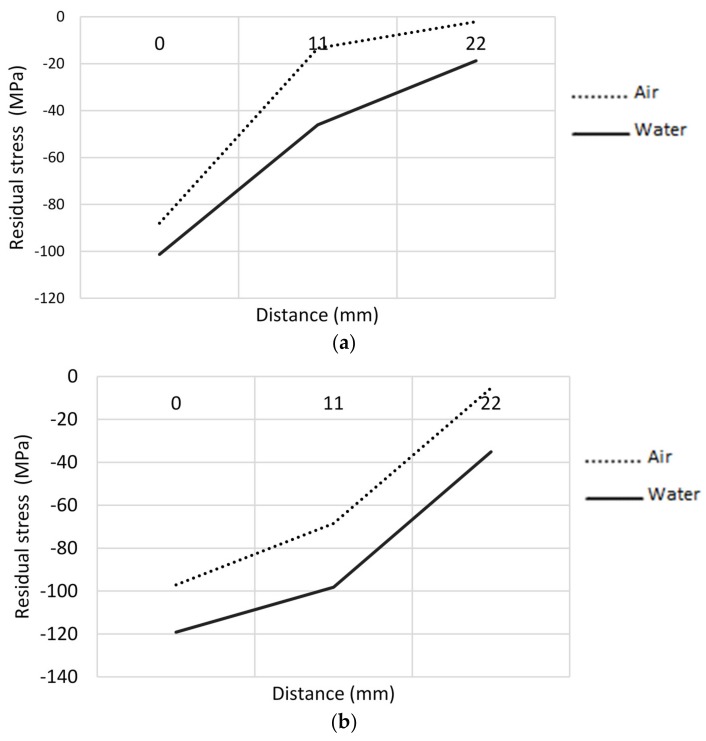
Residual stress obtained from the theoretical model MEF for a leg of (**a**) 3 mm, (**b**) 5 mm.

As a product of computational modeling, a residual stress profile was obtained for each of the legs of welding and cooling media analyzed. In Figure 6, the residual stress profile is shown for a vessel with a leg of five millimeters, where the zero position indicates the location of the weld toe. It was found that the modeled residual stresses were compressive-typed increasing in the closeness of the weld bead, and its magnitude is directly related to the intensity of the cooling medium and the size of the bead. Water is a more intense cooling medium, introducing a rate of cooling in the initial range analyzed of −112 °C/s, and a residual stress at the weld toe for a leg of 5 mm equal to −119 MPa. On the other hand, it was found that large legs induce higher residual stresses, prompted by the need for a greater heat input to the board and greater three-dimensional restriction to thermal contraction. 

**Figure 7:**
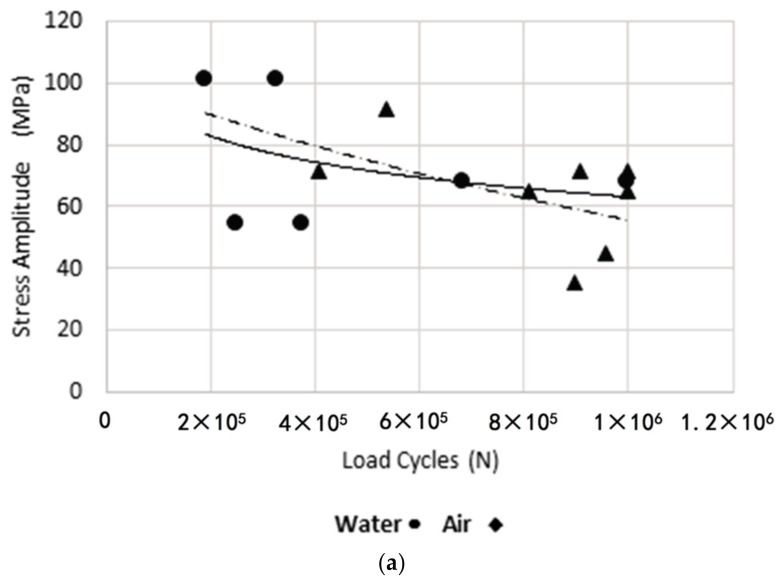

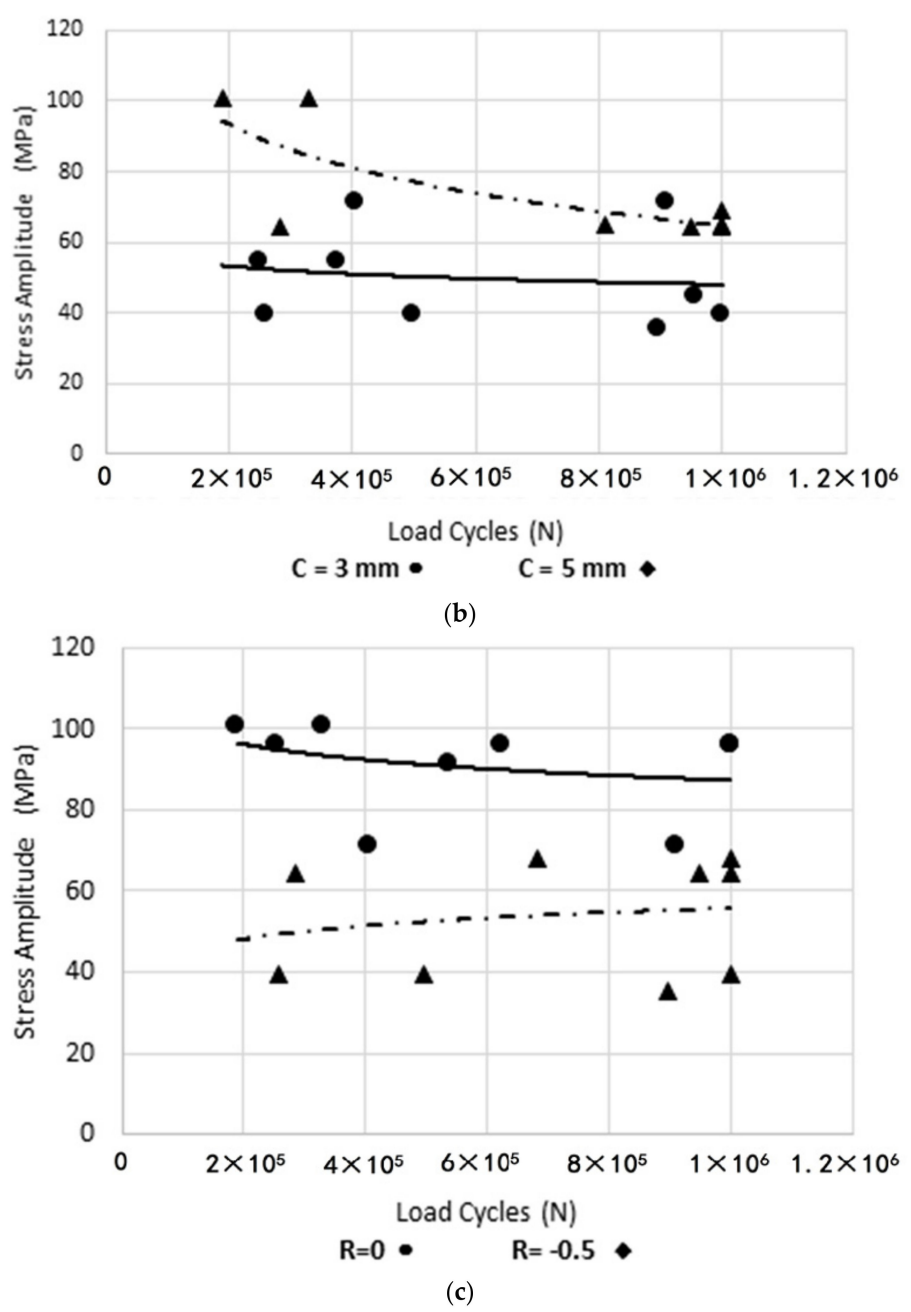
Diagrams Stress—Number of Cycles. (**a**) For water and air; (**b**) For a weld size leg 3 mm and 5 mm; (**c**) For a load ratios 0 and –0.5.

Using the axial fatigue machine, stress-life tests were carried out for the specimens under study. The experimental results for the different cooling media and welding legs are shown below, in Figure 7a. It is observed that more severe cooling media reduce fatigue life. In Figure 7b, it is observed that the size of the leg did not considerably affect the life of the specimens. In Figure 7c, it is observed that the more tensile load ratios minimize the life of the specimens.

The behavior of the weld magnification factor M_k_ in the presence of residual stresses was evaluated analytically. Weld magnification factors M_k_ obtained with the presence of a residual stress field have been appointed in the present research. This allows for making a distinction on this factor in the sense that it involves the effect of the residual stress field in the calculation of the stress intensity factor. The expression (9) is used in the calculation of M_k_ in function of the dimensionless crack depth (a/T) and the possible combinations between weld size (leg) and the cooling medium used, being:

a: Semi-minor axis (mm).
T: Plate Thickness (mm).

**Figure 8:**
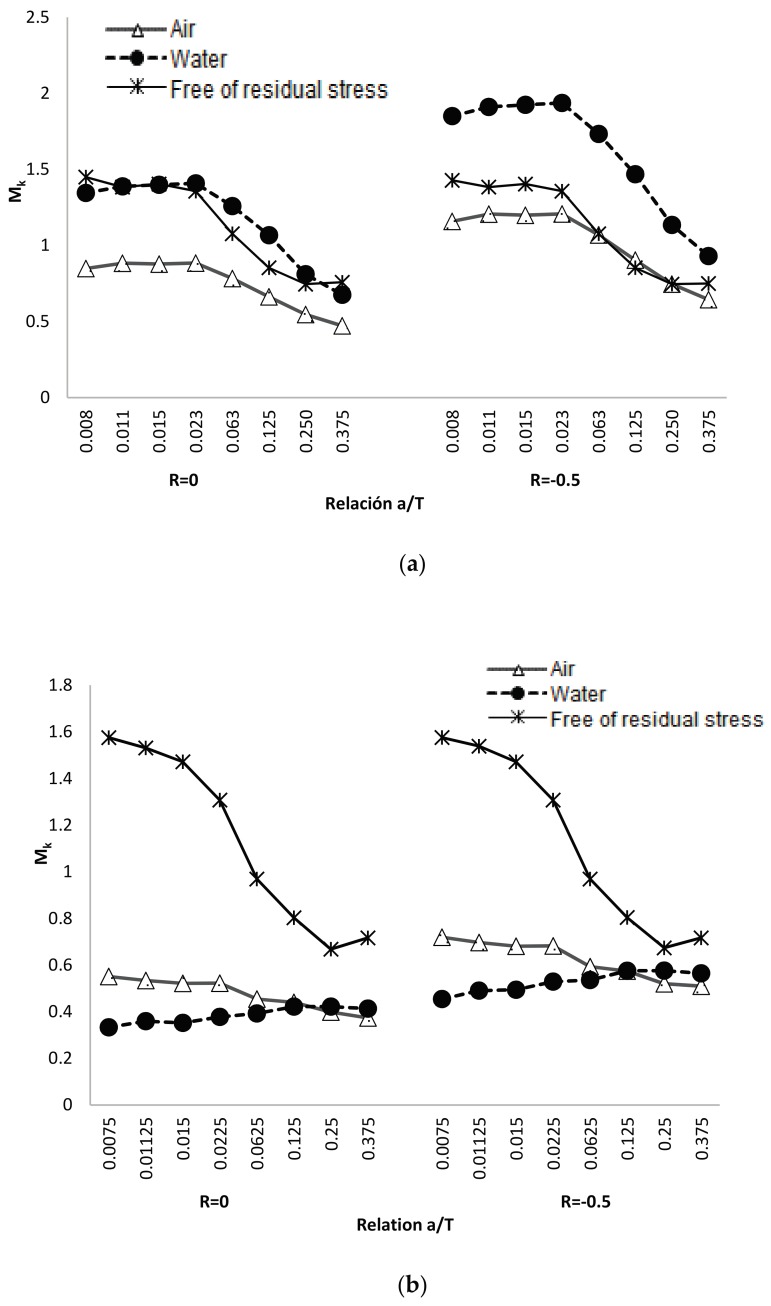
Modified weld magnification factor M_k_ to crack at the weld toe: (**a**) weld size (leg) of 3 mm and (**b**) weld size (leg) of 5 mm.

In Table 6 the values obtained for M_k_ are shown for the different relationships of load rate, type of cooling, and weld size of the study object. The behavior of the modified M_k_ factors, in function of the dimensionless size of crack, is shown in Figure 8 for the two sizes of legs analyzed. 

**Figure 9:**
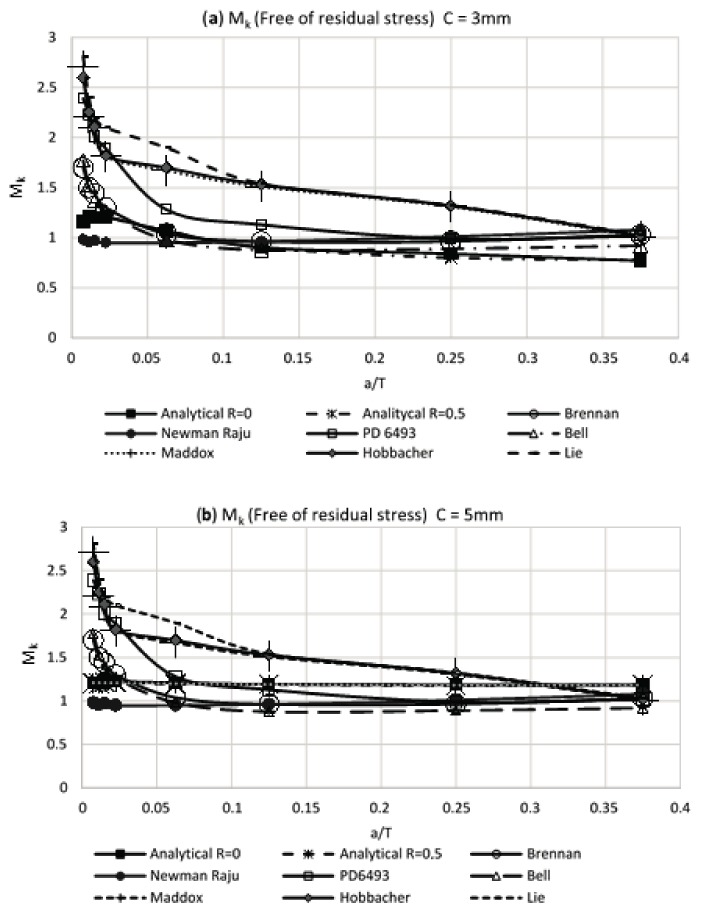
Weld magnification factor M_k_ without residual stress vs. results of other researchers: (**a**) weld size of 3 mm and (**b**) weld size of 5 mm.

The theoretical obtained results in the present work for the modified weld magnification factor M_k_, for condition of free stresses, were compared with the results that were obtained by other researchers [9,10,11,12,13,15,16,17]. In Figure 9, results for the weld magnification factor are shown, for the case of a crack at the weld toe and without residual stress, and verifies the correspondence of the developed numerical model with the results obtained by other researchers. The analytical results obtained involve several weld sizes and load rates.

**Figure 10:**
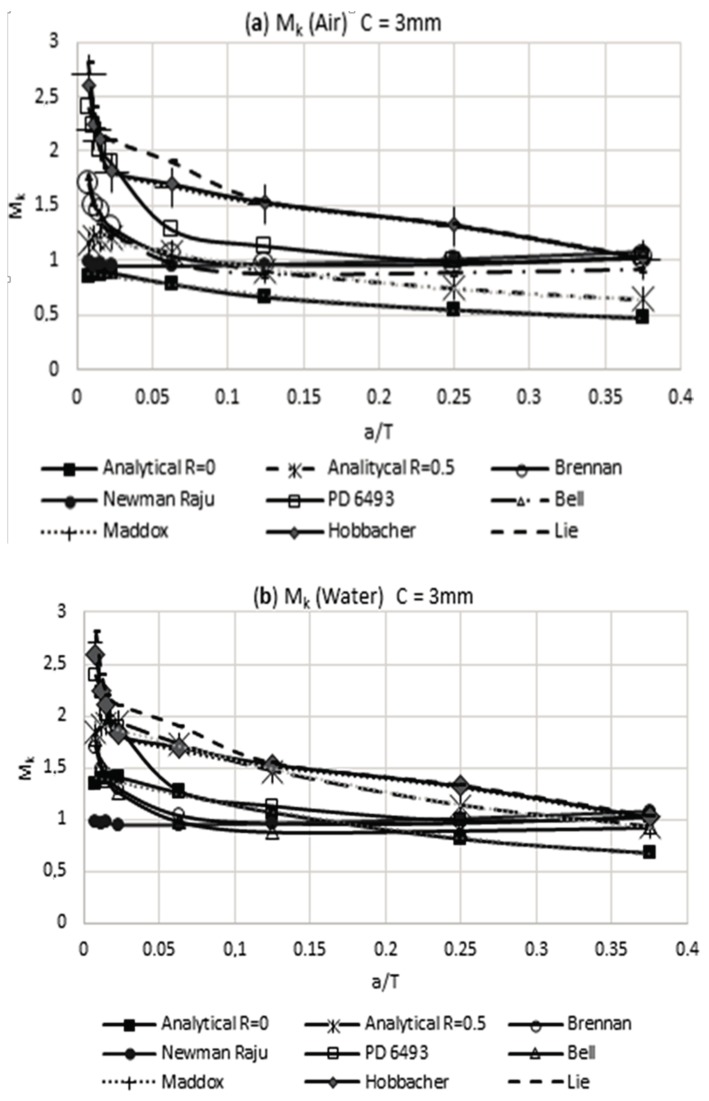
Modified weld magnification factor M_k_ with the presence of residual stress vs. other researchers: (**a**) air and weld size of 3 mm and (**b**) water and weld size of 3 mm.

**Figure 11:**
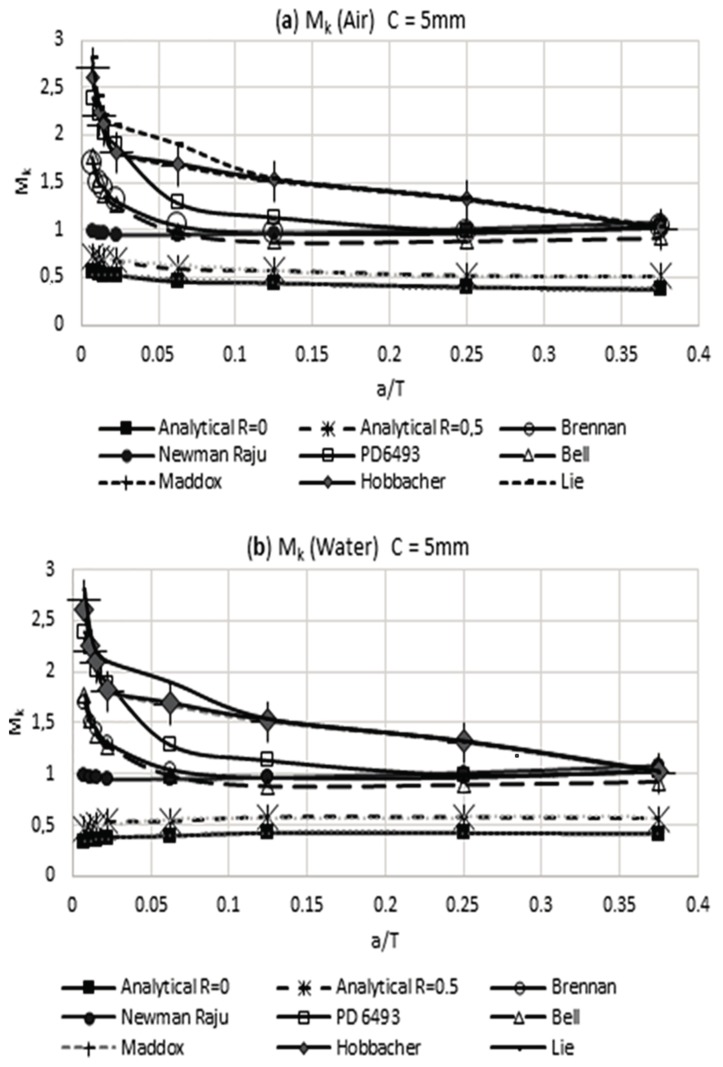
Modified weld magnification factor M_k_ with the presence of residual stress vs other researchers: (**a**) air and weld size of 5 mm and (**b**) water and weld size of 5 mm.

In Figure 9a,b, it can be noted that the weld magnification factor M_k_ is independent from the load rate and has a similar behavior to that proposed by other researchers. Using the obtained information in Table 6 for the modified weld magnification factor M_k_, including the effect of the residual stress reached by the air and water cooling media, it is possible to make a comparison with the results that were obtained by other researchers. This comparison of results is shown in Figure 10 and Figure 11. The observed trend with the modified weld magnification factor is to markedly diminish in function of the post weld cooling medium intensity, for the range of relative size of crack a/T > 0.1. This behavior is related to the coupled benefits of the residual compressive stresses that arise during the post-welding cooling for the region where the modeled crack occurs in the present work. 

With the results that were obtained for the weld magnification factor, a regression analysis is carried out to obtain analytical equations that relate to the dimensionless size of the crack. Table 7 shows the expressions of M_k(a/T)_ for the free condition of residual stresses. In Table 8, the expressions of M_k(a/T_) are shown for the condition of post-weld cooling in calm air. Finally, Table 9 shows the expressions of M_k(a/T)_ for the condition of post-weld cooling in water (in the equations Ω = a/T). These analytical expressions are particularly useful to establish models for the prediction of fatigue crack propagation and the design of a life management program for welded structures of the studied type. 

## 4. Conclusions

We conducted a theoretical experimental study about the behavior of fatigue in welded joints with cruciform geometry. A 3D simulation model of the welded joint was used throughout the finite element method where several features were introduced, such as the superficial natural undulation of the weld bead and established, and the mechanical properties of the fusion zone, the heat affected zone and the base material, respectively. In addition, a residual stress field was introduced for the welded joint and the surrounding region, which emulates the one obtained experimentally. In the computational simulation of the superficial semi-elliptical crack at the weld toe, a convergence of the model for 405428 nodes, with a computational cost in central processing unit (CPU) time of 2680 s for each iteration, was reached.

It was determined that the residual stresses are of compression higher for the more intense cooling medium (water). In addition, in Figure 6, it can be noted that larger weld size induces greater residual stresses, prompted by the need for a greater heat input to the joints and to the greater three-dimensional restriction to a thermal contraction of the weld joint. Fatigue tests indicate that more severe cooling media minimizing the life of the welding specimens in the same way as the more tensile load ratios. It is observed that the specimens mainly failed in the weld toe.

A unique finding of the present work is the reaching of analytical expressions obtained by the weld magnification factor M_k_ for two sizes of the weld and two post welding cooling media. The analytical equations obtained consider the residual stresses induced by these two post welding cooling mediums. The analytical expressions for M_k_ in the present research have good correspondence with the obtained results by other authors, in the case of welded joints without residual stresses. These expressions can improve the calculation codes, testing standards, and the structural integrity of welded joints verification. It can be noted that the observed trend with the modified weld magnification factor is to markedly diminish, in function of the intensity of post welding cooling medium for a dimensionless crack size below a/T < 0.1. This behavior is related to the coupled benefits of the residual compressive stresses that arise during the post-welding cooling for the assessment region for the crack–type studied. 

## Figures and Tables

**Table 1:** Main characteristics of the welding procedures used.

Weld Size (Leg)	Diameter of the Electrode E-6013	Electrical Parameters	Forward Speed
3 mm	3/32″	102 volts, 72 A CD	20 cm/min
5 mm	1/8″	71.2 volts, 98 A CD	20 cm/min

**Table 2:** Variables for manufacturing.

Weld Size (Leg) C	Cooling Medium
3 mm	Air calm
5 mm	Water

**Table 3:** Parameters of the equipment.

Parameters	Magnitude	Unit
Maximum load	900	Kgf
Frequency of operation	12	Hz
Engine power	3	Hp
Nominal Motor Amperage	8.6	A
Supply voltage	206	v
Amperage at the Operation point	8.3	A
Load application cycles per Hour	8200	Cycles
Diameter Drive Pulley in	6	In
Diameter Pulley Driven in	12	In
Transmission Ratio	0.5	-
Motor Speed rpm @ 60 Hz	3445	Rpm

**Table 4:** Crack sizes of semielliptical section.

Semi-Major Axis (c) (mm)	Semi-Minor Axis (a) (mm)
0.15	0.06
0.23	0.09
0.30	0.12
0.45	0.18
1.25	0.50
2.50	1.00
5.00	2.00
7.50	3.00

**Table 5:** Nominal and alternant stresses for the fatigue test.

Load Rate (R = Pmin/Pmax)	Nominal Stress $\sigma_{\mathrm{nom}}$ (MPa)	Alternant Stress $\sigma_{\mathrm{alt}}$ (MPa) C = 3 mm	Alternant Stress $\sigma_{\mathrm{alt}}$ (MPa) C = 5 mm
0	55.2	71.3	100.9
−0.5	36.8	35.5	64.6

**Table 6:** Modified weld magnification factor M_k_ to crack at the weld toe: (a) weld size (leg) of 3 mm and (b) weld size (leg) of 5 mm.

Load Rate (R)	a/T	Cooling Air	Cooling Water	Free of Residual Stress
(a) Modified weld magnification factor M_k_ for weld size (leg) of 3 mm.				
0	0.008	0.848	1.345	1.449
	0.011	0.884	1.389	1.384
	0.015	0.878	1.399	1.404
	0.023	0.884	1.408	1.357
	0.063	0.783	1.260	1.077
	0.125	0.662	1.067	0.853
	0.250	0.547	0.811	0.746
	0.375	0.472	0.676	0.758
−0.5	0.008	1.159	1.851	1.428
	0.011	1.206	1.911	1.384
	0.015	1.199	1.925	1.404
	0.023	1.207	1.937	1.357
	0.063	1.069	1.733	1.077
	0.125	0.904	1.469	0.854
	0.250	0.746	1.135	0.746
	0.375	0.644	0.930	0.750
(b) Modified weld magnification factor M_k_ for weld size (leg) of 5 mm.				
0	0.008	0.551	0.333	1.575
	0.011	0.534	0.359	1.531
	0.015	0.522	0.352	1.471
	0.023	0.522	0.378	1.307
	0.063	0.454	0.393	0.969
	0.125	0.440	0.422	0.803
	0.250	0.398	0.422	0.668
	0.375	0.373	0.413	0.717
−0.5	0.008	0.719	0.455	1.575
	0.011	0.697	0.491	1.538
	0.015	0.681	0.494	1.472
	0.023	0.682	0.529	1.307
	0.063	0.593	0.536	0.969
	0.125	0.574	0.576	0.804
	0.250	0.520	0.576	0.674
	0.375	0.509	0.564	0.717

**Table 7:** Adjusted expressions for the weld magnification factor for the free condition of residual stresses.

Weld Size (Leg)	
3 mm	5 mm
M_k_ = 0.0077Ω^3^ − 0.1121Ω^2^ + 0.351Ω + 1.1592	M_k_ = 0.0101Ω^3^ – 0.1361Ω^2^ + 0.3671Ω + 1.3123

Valid for: 0.02 ≤ Ω ≤ 0.33.

**Table 8:** Adjusted expressions for the modified weld magnification factor M_k_ for the condition of post weld cooling in calm air.

Load Rate (R)	Weld Size	
	3 mm	5 mm
0	M_k_ = 2.5098Ω^2^ − 2.0772Ω + 0.9002	M_k_ = 1.5822Ω^2^ − 1.0261Ω + 0.5416
−0.5	M_k_ = 4.5708Ω^2^ − 3.7817Ω + 1.6383	M_k_ = 3.2069Ω^2^ − 1.8877Ω + 0.9443

**Table 9:** Adjusted expressions for the modified weld magnification factor M_k_ for the condition of post weld cooling in water.

Load Rate (R)	Weld Size	
	3 mm	5 mm
0	M_k_ = 3.2442Ω^2^ − 3.2442Ω + 1.4311	M_k_ = 7.0363Ω^3^ − 5.4379Ω^2^ + 1.2513Ω + 0.338
−0.5	M_k_ = 5.5871Ω^2^ − 5.7921Ω + 2.6206	M_k_ = −117.47Ω^4^ + 97.992Ω^3^ − 28.427Ω^2^ + 3.4545Ω + 0.6097
